# Improvement in Knowledge Level of Associate Degree Nursing Students in Zarqa University College Regarding Care for Patients With Indwelling Urinary Catheters After Joining an Educational Session

**DOI:** 10.5539/gjhs.v7n6p39

**Published:** 2015-03-25

**Authors:** Yahya W. Najjar, Maha T. Hdaib, Siham M. Al-Momany

**Affiliations:** 1 Al-Balqa’a Applied University, Zarqa University College, Zarqa, Jordan

**Keywords:** catheter, education, indwelling, patient care

## Abstract

**Background:**

Care for patients with indwelling urinary catheter is one of the most important factors in preventing problems associated with indwelling urinary catheters. Caregiver education about caring of indwelling urinary catheters is important the same as patient education to prevent urinary catheters problems.

**Methods:**

A pre test post test control group design was used with nursing students of Zarqa University College. Data were collected through self-administered questionnaire.

**Results:**

Experimental group did not do better in the posttest than the control group after they joined an educational session about caring for patients with indwelling urinary catheter. Level of student (first year vs. second year) has been identified as a covariate that may have distorted the results.

**Conclusion:**

One educational session is insufficient to change level of knowledge regarding caring for patients with indwelling urinary catheter, in addition to considering the level of nursing student when conducting the educational session.

## 1. Introduction

Approximately 4 million people have an indwelling urinary catheter (IUC) inserted each year (Adib-Hajbaghery & Aghajani, 2009). Urinary tract infection (UTI) is the most important adverse outcome of urinary catheter use, 80% of hospital acquired UTI(s) are attributable to an IUC (Anderson et al., 2008). Catheter use is also associated with negative outcomes other than infection, including nonbacterial urethral inflammation, urethral strictures, mechanical trauma (Anderson et al., 2008), bacteremia, leakage and recurrent blockage (Adib-Hajbaghery & Aghajani, 2009). Complications associated with long-term catheterization are bacteriuria and UTI, encrustation and subsequent blockage (Madigan & Neff, 2003). UTI(s) have been shown to increase length of stay, hospital cost, and mortality (Rosenthal, Guzman, & Safdar, 2004).

Care for patient with IUC is an important element to prevent problems associated with indwelling IUC. Care for patient with IUC was described by Lo et al. (2008) which includes properly securing indwelling catheters after insertion, maintaining a sterile, continuously closed drainage system, not disconnecting the catheter and drainage tube unless the catheter must be irrigated, obtaining urine samples for culturing by aspirating urine from the sampling port, maintaining unobstructed urine flow, emptying the collecting bag regularly, keeping the collecting bag below the level of the bladder at all times, and cleaning the meatal area (area of urethral meatus) without using antiseptics. Many urinary catheter-associated problems are preventable by teaching the patients about simple measures such as hand washing, maintaining a closed system, securing the catheter, drinking enough fluids and properly positioning the drainage bag (Adib-Hajbaghery & Aghajani, 2009). Use of antiseptic or antimicrobial solutions or ointments as part of routine meatal care does not reduce the incidence of bacteriuria or catheter associated urinary tract infection (CAUTI), so regular cleansing of the meatus as part of routine perineal hygiene without using antiseptics or antimicrobials may reduce the incidence of CAUTI, especially in patients with fecal incontinence (Willson et al., 2009). The use of external male catheter (condom) has been shown to reduce bacteriuria, CAUTI, and death in male patients (Saint et al., 2006). Slater (2011) reported that regular check up of IUC and drainage system and patient education regarding IUC care are important in the safe management of caring for individuals with long-term IUC(s).

Despite the strong link between urinary catheters and subsequent UTI, A national study conducted in the United States revealed that no single strategy was widely used for the prevention of nosocomial UTI (Saint et al., 2008). Intermittent urinary catheterization when required in managing elderly female urinary retention is superior to using IUC as IUC would hinder rehabilitation and adversely affect patient quality of life (Tang, Kwok, Hui & Woo, 2006). Adib-Hajbaghery and Aghajani (2009) reported that the quality of care for patients with IUC(s) was low regarding patient preparation for urinary catheterization, patient education, and post catheterization care. Many of the indications for urinary catheterization are considered inappropriate such as to replace nursing care for urinary incontinence and prolonged postoperative periods without appropriate indications for urinary catheter use (Gould, Umscheid, Agarwal, Kuntz & Pegues, 2010). For this reason, staff education about indwelling urinary catheter management (i.e.: techniques for catheterization, catheter care, and technique of catheter removal) and prevention of CAUTI, combined with regular feedback about occurrence rates, reduces the incidence of bacteriuria and CAUTI (Willson et al., 2009). Mamatha (2005) concluded that structured teaching program is an effective method of improving knowledge and skills of staff nurses regarding indwelling catheter care in order to prevent the complications associated with IUC.

Because health care providers do not concentrate on preventing measures of IUC associated problems (Adib-Hajbaghery & Aghajani, 2009), and no studies specific to care for patient with IUC were conducted in Jordan, and no previously proven method to increase knowledge level about caring for patient with IUC, the study purposed to assess knowledge level of associate degree nursing students in Al-Balqa’a Applied University/Zarqa University College-regarding care for patients with IUC and to determine whether their knowledge level differ after they join a session of care for patient with IUC.

## 2. Methodology

### 2.1 Design

Pre-test post-test control group design (Polit & Beck, 2012), in which one group assigned to join an educational session about caring for patient with IUC, and the other group kept as a control group receiving an educational session about a different educational session (regular educational session according to the course schedule for fundamental of nursing course).

### 2.2 Sample

The entire population (i.e. associate degree nursing students in Zarqa University College during the academic year 2013/2014) equals to 70 students, all of them were selected to participate in the study and they were acknowledged to participate freely (autonomously) or to withdraw at any time during the study.

### 2.3 Settings and Procedure

The participants in the study group had a pretest (paper and pencil test) about caring for patient with IUC then they joined an educational 40-minute session on 14^th^ May 2014 about caring for patient with IUC (see appendix A), then after 2 weeks (on 28^th^ May 2014) they were asked to complete the same version of the paper and pencil test they had in the pretest. The same approach (pretest, joining an educational session, and posttest) was applied to the control group.

### 2.4 Data Collection

The instrument used in this study to assess the knowledge for administering IM injection was a self - administered questionnaire designed for the purpose of this study and developed from the protocols and guidelines provided by of Lo et al. (2008), Adib-Hajbaghery and Aghajani (2009) and Willson et al. (2009), which was validated by three clinical and academic personnel (see acknowledgment section). The questionnaire (test) contained fourteen yes/no questions written in Arabic reflecting the knowledge of participant for care of patient with IUC (see Appendix B). The maximum score for the test was 14, with higher scores indicating more knowledge about caring for patient with IUC. On the posttest, an increase in the scores of participants will indicate an effectiveness of the teaching session of increasing knowledge about caring for patient with IUC.

### 2.5 Data Analysis

First data will be screened for missing data and unexpected responses and treated with appropriate techniques. Means, standard deviations, and ranges for overall score for each participant will be calculated. For comparison between the experimental and control groups, a parametric test (e.g.: t-test) will be used if the data were normally distributed. Non-parametric tests, such as Chi-square or Mann-Whitney U will be used if data were not normally distributed to detect the differences between the both groups in the pretest and the posttest. All statistical analyses will be calculated using SPSS program version 21 (George & Mallery, 2003). α level of statistical significance was determined at 0.05

## 3. Results

Participants were randomly allocated (using the table of random numbers) into both groups and were distributed as the following: 33 participants in control group versus 33 participants in experimental group (4 students decided not to participate in the study).

Response rate in pre test was 94% (66 out of 70 participants completed the pre test), while response rate in post test was 88% (58 out of 66 participants completed the post test), which made unequal number of participants in both control and experimental groups even though they were distributed equally in both groups prior to data collection. 37(63.8%) participants were first year nursing students, whereas 21 (36.2%) participants were second year nursing students.

Only slight changes were observed in the scores of posttest compared to the scores of pretest for the experimental group (M: 11.57, SD: 1.85 in post test compared to M: 9.96 SD: 2.06 in pre test). The same applies on the control group (Table 1).

**Table 1:** Pre- and post test scores of participants (*n* = 58)

	Range	*M* (*SD*)
Pretest scores (overall)	6 to 14	10.41 (1.83)
Control group	8 to 13	10.83 (1.49)
Experimental group	6 to 14	9.96 (2.06)
Posttest scores (overall)	6 to 14	11.36 (1.72)
Control group	7 to 13	11.17 (1.60)
Experimental group	6 to 14	11.57 (1.85)

An independent sample t-test (Table 2) was performed to assess whether there was difference between experimental and control group. Data were screened for missing data and case deletion method was used for participants who had no responses in post test. The data of results for pre test and post test were approximately normally distributed and not judged to have seriously enough departure from normality to require the use of a nonparametric test. The assumption of homogeneity of variance was assessed by the Levene’s test: *F* = 1.81, *p* = .18 for pre test results. *F* = .12, *p* = .73 for post test results; this indicated no significant violation of the equal variance assumption; therefore, the pooled variances versions of the *t* tests were used. The results of pre test did not differ significantly between control and experimental group: *t*(56) = 1.85, *p* = .17, whereas results in post test differed significantly: *t*(56) = 3.95, *p* = .02. This means that experimental group did better in the post test than the control group.

**Table 2:** Independent samples t-test results for control and experimental group

Groups	*N*	*M*	*SD*	*t*	*df*	*p*
Pretest						
Control	30	10.83	1.49	1.85	56	.07
Experimental	28	9.96	2.06			
Posttest						
Control	30	11.17	1.60	-.89	56	.38
Experimental	28	11.57	1.85			

A paired sample t-test (Table 3) was conducted to compare the difference between results of the experimental group in both pre test and post test. As the results of both pre test and post test were approximately normally distributed, no departure from normality was judged serious enough to require the use of nonparametric test. Pre test and post test results were significantly correlated to each other (*r* = .66, *p* < .001), and there was significant difference between pre test and post test results: *t*(27) = -5.26, *p* < .001. These results suggest that results of post test differed significantly from results of pre test in the experimental group and the experimental group participants did better in the posttest than in the pretest. On the other hand, the results for comparison group in pretest and posttest did not differ significantly (*t*(29) = -1.17, *p* = .25).

**Table 3:** Paired sample t-test results for the experimental group in both pre test and post test

Groups	*N*	*M*	*SD*	*t*	*df*	*p*
Experimental (pretest)	28	9.96	2.06	-5.26	27	<.001
Experimental (posttest)	28	11.57	1.85			

Based on the previous results (i.e.: statistically significant paired sample t-test and non-statistically significant independent samples t-test), a confounding variable may have distorted the results of t-tests, so analysis of covariance (ANCOVA) was conducted to reveal the effect of participant level on the results of the posttest (i.e.: whether level of participant as a first year or second year student affected the results of the posttest). As shown in table 4, the results revealed a significant interaction between level of student and the results of posttest scores (*F*(9,48) = 5.98, *p* < .001), so it can be judged that level of student affected the results of posttest scores for the experimental group.

**Table 4:** Analysis of covariance for between- subjects effect

Covariate	Type III sum of squares	*df*	Mean square	*F*	*P*
Results of posttest * Level of student	89.54	9	9.95	5.98	<.001
Error	79.86	48	1.66		
Total	7657.00	58			

## 4. Discussion

The study purposes were to assess knowledge level of associate degree nursing students regarding caring for patient with IUC and whether their knowledge level change after joining the educational session about caring for patient with IUC. The results obtained from this study have shown that knowledge level of students has not been changed dramatically in posttest results (overall mean of scores changed from 10.41 in pretest to 11.36 in posttest). Among the possible causes for these results is inability of one educational session to make considerable change in the knowledge level of students. Grol (2001) concluded that a program to implement guidelines for clinical practice should be well designed, well prepared, and preferably pilot tested before use. Adib-Hajbaghery and Aghajani (2009) have emphasized on the need for continuing education and reinforcement of the required behaviors in nursing and medical staff involved in IUC insertion to reduce the risk of developing UTI. The same point was addressed by Rosenthal, Guzman, and Safdar (2004) that implementation of education combined with performance feedback is necessary to increased compliance with guidelines for caring of patient with IUC and subsequent reduction in CAUTI rates, similarly as what Mamatha (2005) concluded that structured teaching program is an effective method of improving knowledge and skills of staff nurses regarding indwelling catheter care in order to prevent the complications associated with IUC, so the combination of continuing education, and performance feedback and reinforcement is necessary to increase knowledge level and skills of nursing students in caring for patient with IUC.

The results have also shown no significant difference between the control and the experimental group in the posttest results, and there was a significant interaction between posttest scores and level of student in the university (first year vs. second year). Second year students know more about caring for patient with IUC due to their practical training in teaching hospitals, and so what was added to their knowledge in the educational session is little compared with what was added to the knowledge of the first year students. Tobias (1994) concluded that prior knowledge about a specific topic raises more interest in learning what is new about that topic, a point that has been reported by Valliani, Ahmed, Saleem, Mirza, and Ejaz (2011) that old nurses are trained well and are doing better than the fresh medical graduates. However, Adib-Hajbaghery and Aghajani (2009) have reported that there was inappropriate or lack of communication between caregiver and patient regarding using IUC, a point that indicates lack of knowledge or the application of traditional and irrationalized health care task. To plan for a change in clinical practice, characteristics of individual professionals and interpersonal factors should be addressed (Grol & Wensing, 2004). So presence of second year nursing students might have impacted the results obtained from this study because of their traditional learning and their prior knowledge gained during their clinical training in hospitals that might be irrationalized or not evidence-based, despite their good and holistic performance in providing nursing care.

## 5. Conclusion and Recommendations

In conclusion, joining one educational session is insufficient to increase knowledge level of associate degree nursing students regarding caring for patient with IUC, so it is recommended to include more than one educational session combined with reinforcement of the required behaviors while conducting educational programs about caring for patient with IUC. Furthermore, nursing students should be observed for their performance if will change from traditional and non-evidence based practices to the evidence- based and safe practices.

Level of student during university education should be addressed in future studies because of its confounding effects on the results of this study, a factorial design or repeated measure design (Polit & Beck, 2012) may isolate the effect of student level in both the experimental and control groups from the results obtained or to measure the change in knowledge level at different times.

## Appendices

Appendix A

session plan for caring for patient with IUC.

**Title:** nursing care of indwelling urinary catheter

**Day and Date:** Tuesday: 14/5/2014 **Time:** 40 minutes

**Objectives:** by the end of the session, participants will be able to:

1) Understand the need and rationale for parineal and urethral care for patient with indwelling urinary catheter.
2) Acknowledge the principles of care for urinary drainage system.
3) Know key assessment points for patient with indwelling urinary catheter.

**Table:**

Stages	Time	Subjects	Methods	Trainers’ activities	Participants’ activities	Aids
Introduction.	10.	Title. Session objectives. Pre test.	Lecturing. Multiple choice and true/false questions	Mentioning title and objectives. Conducting the test.	Listening. Answering the questions.	White board.

Content	10 5 5 5	Perineal and urethral care Care of drainage system Obtaining urine specimens Patient assessment	Lecturing. Discussion. Lecturing Brain storming. Lecturing.	Explaining rationale, need and frequency of parineal care. Facilitating discussion. Mentioning principles of urinary drainage system care Asking participants how can you obtain urine specimen from catheter or drainage system safely? Mentioning and clarifying points of continuous and regular assessment of patient with indwelling urinary catheter.	Listening. Joining the discussion. Listening Answering the question. Listening	Data show Data show. White board. Data show. Data show

Revision, summary, and evaluation	5	Revising and evaluating the session. Post test.*	true/false questions.*	Asking students about benefits obtained from the session. Conducting the test.*	Providing feedback. Answering the questions.*	

* : post test is to be conducted later on after 2 weeks.

Appendix B

The self- administered questionnaire about caring for patient with IUC.

Participant name: __________________________ gender: _________ level: _______

____________________________________________________________________________________

Answer the following statements as **yes** or **no**:

1. To prevent urinary catheter from being pulled or kinked, it should be taped to the inner aspect of the thigh in women or to lower abdomen in men. _______
2. Water soluble lubricant may be applied to the urethral meatus to decrease irritation. _______
3. The perineal area of women should be cleaned from back to front._______
4. Urine bag and tubing should be above the level of the urinary bladder.________
5. Urinary drainage system should be a closed system._______
6. Obtaining urine specimen for culture can be obtained directly from the urine bag._______
7. No tubing should loops hang below the level of the bladder. ________
8. You can clean the perineal area and catheter using soap and water._____
9. Urine bag should be emptied every 8 hours.________
10. Urine bag can be attached to the side rails.________
11. Sterile technique must be maintained during opening the urinary drainage system. ________
12. Patient with urinary catheter and drainage system can drink up to 3 liters of fluids daily. ________
13. Color and turbidity of urine can be noticed by looking at urine in the urine bag only. ________
14. Cleaning the patient’s perineal and urethral meatus should be at least once every week. ________
